# The effect of hearing impairment and social participation on depressive symptoms in older adults: a cross-lagged analysis

**DOI:** 10.3389/fncom.2023.1240587

**Published:** 2023-08-08

**Authors:** Hejun Zhao, Xinying Wang, Yuhao Shi

**Affiliations:** ^1^School of Humanities, Henan Kaifeng College of Science Technology and Communication, Kaifeng, Henan, China; ^2^School of Psychology, Henan University, Kaifeng, Henan, China; ^3^The First Affiliated Hospital of Tsinghua University, Beijing, China

**Keywords:** hearing impairment, social participation, depressive symptoms, cross-lagged analysis, CHARLS data

## Abstract

**Objective:**

This study aimed to investigate the relationship between hearing impairment, depressive symptoms, and social participation in older adults.

**Methods:**

The study used data from the China Health and Retirement Longitudinal Study (CHARLS) in 2013 and 2018, which included 3,980 samples. The analysis was conducted using cross-lagged structural equation modeling with SPSS 23.0 and Mplus 7.4.

**Results:**

The findings show that from 2013 to 2018, older people had significantly more hearing impairment and depressive symptoms and significantly less social participation. Hearing impairment was a significant negative predictor of social participation, and older adults with hearing impairment were less likely to participate in social activities. In addition, there may be a bidirectional relationship between hearing impairment and depressive symptoms, with both being positive predictors of each other. Finally, the study found that social participation played an important mediating role in the relationship between hearing impairment and depressive symptoms.

**Conclusion:**

The study’s findings highlight the complex interplay between hearing impairment, social participation, and depressive symptoms in older adults. Therefore, it is important to intervene promptly when hearing impairment is detected in the elderly; pay attention to patient guidance and comfort for the elderly with hearing impairment, give them positive psychological support, encourage them to get out of the house and participate in more social activities to avoid depressive symptoms. The study’s results may inform the development of targeted interventions to address the mental health needs of older adults with hearing impairment.

## 1. Introduction

Based on the most recent census data, the proportion of senior citizens in China currently comprises 18.7% of the total population, indicating that the nation has transitioned into an aging society, with a growing number of elderly individuals and an increasing life expectancy (Cao and He, 2022). The sensory function of older people gradually declines with age, with hearing loss being the most pronounced. In 2018 World Health Organization statistics show that approximately one third of people over 65 years of age have moderate or greater hearing loss (Brewster et al., 2021). Hearing loss is one of the three most common health conditions in older people, second only to heart disease and arthritis (Ye et al., 2020). In China, about 11% of people over 60 years of age suffer from hearing impairment (Xie et al., 2021). Hearing impairment affects not only daily communication, cognitive ability, quality of life and more chronic co-morbidities, but also mental disorders including depression, anxiety and schizophrenia in older people. The mechanism may be that hearing impairment hinders information exchange and social participation, which in turn impairs physical activity, increases psychological burden and affects the psychological wellbeing of older people (Brewster et al., 2021). Consequently, enhancing the health of the elderly become a widespread societal concern, warranting further investigation and attention.

After entering the old age stage, the physical functions and nervous system of individuals will degenerate to a certain degree, and the physical and cognitive aging will bring many problems to the elderly, such as generating cognitive impairment, communication impairment, and psychological impairment, which seriously affect the normal life of the elderly, the most common of which is hearing impairment. Senile hearing impairment refers to the decline in auditory function caused by aging and degenerative lesions of the auditory organs (Chou, 2008), Hearing changes can lead to reduced sensitivity to higher frequency sounds in older adults, which may also indirectly affect their speech comprehension, especially in poorer listening environments (e.g., when there is background noise or poor telephone signals). Hearing impairment not only affects older people’s daily communication, cognitive abilities, quality of life and more chronic co-morbidities, but also mental disorders including depression, anxiety and schizophrenia. The mechanism may be that hearing impairment hinders information exchange and social participation, which in turn impairs physical activity, increases psychological burden and affects the mental health of older people (Ye et al., 2020). The deleterious impact of hearing impairment on the physical and mental wellbeing of older adults is widely recognized. Drawing upon stress and coping theory, it may be posited that such physical health challenges can be construed as psychological stressors (Holmes and Rahe, 1967), with hearing impairment potentially constituting a persistent stressor that can erode the psychological resilience of older adults. A growing body of literature suggests that hearing impairment among older adults is linked to a range of negative outcomes, including decreased wellbeing, lower quality of life, limited social engagement, cognitive decline, and heightened negative affect such as anxiety, depression, and loneliness (Bourque et al., 2007; Bute et al., 2007). However, most of these studies have been cross-sectional in nature and have only partially elucidated the mechanisms underlying the effects of hearing impairment on the mental health of older adults. Therefore, to promote healthy aging and safeguard the health of older adults, it is imperative to conduct research that comprehensively investigates the psychological mechanisms through which hearing impairment affects the wellbeing and mental health of normally aging older adults, so that effective measures can be taken to improve the wellbeing and mental health of the elderly.

In terms of neurobiological mechanisms, it is possible that auditory decline may lead to reduced stimulation of central auditory pathways, which in turn may lead to atrophy of frontal brain regions and ultimately cognitive or executive dysfunction, increasing the risk of depression (Gao et al., 2020). Depressive symptoms are a type of affective psychiatric disorder that is marked by a constellation of symptoms including a pervasive sense of sadness, reduced motivation, and cognitive slowing, and in more severe cases, may be accompanied by suicidal ideation. The factors influencing depressive symptoms are richly researched, and studies have shown that the factors influencing depressive symptoms in older adults can be divided into: subjective factors, objective factors, and general demographic factors. Subjective factors generally include personality traits, subjective wellbeing, life satisfaction, and long-established coping styles of older adults. Objective factors include personal, family, and social factors. And demographic factors include age and gender (Wu, 2003). The process of aging is accompanied by changes in both physical and mental health, as well as various life events such as changes in family dynamics, loss of close companions, retirement-related financial constraints, and a sense of diminished social roles, all of which can contribute to elevated levels of depressive symptoms among older adults. In addition, existential concerns such as fear of death and other psychological factors may further exacerbate depressive symptoms in this age group, surpassing the levels observed in younger age cohorts. In addition, older adults are excluded from the labor market due to old age, and social changes such as loss of social roles, low status, and reduced social support severely affect older adults’ loneliness and depressive symptoms (Devkota et al., 2019). Depression has emerged as the most prevalent psychological disorder among older adults, affecting approximately 7–10% of this population and up to 50% among those with comorbid somatic illnesses. Its pernicious impact extends beyond the individual to encompass the entire family, causing significant harm and distress (Rong et al., 2020). Hearing impairment disorders, as one of the somatic disorders, seriously affect depressive symptoms in older adults, and the decline in hearing in older adults can induce more severe depressive symptoms. Analyzing longitudinal data on aging in Australia, one study found a significant correlation between hearing and depression (Kiely et al., 2013). However, some scholars have also used data from the China Health and Aging Tracking Survey (CHARLS) and found that hearing loss was not associated with depressive symptoms (Xie et al., 2021). However, some studies have also found that depressive symptoms, as a serious psychological disorder, can trigger somatic diseases, such as cognitive decline, heart disease, hypertension, and other chronic diseases (Wang, 2020). There is debate as to whether hearing status has a significant effect on depression in older people, but most studies suggest that hearing status has a significant effect on depression in older people (Ye et al., 2020). Therefore, depression and hearing impairment may be co-morbid, so this study suggests that depressive symptoms can lead to hearing loss, and hearing loss can trigger more severe depressive symptoms.

Social network theory suggests that social networks influence health by promoting social participation (Gao et al., 2020). Social participation refers to the activities that participants engage in to meet their social needs in social interactions (Levasseur et al., 2008). Social participation as a mode of behavior of participants in the process of social interaction can provide individuals with role identity, companionship and increased social awareness (Gao et al., 2020). Social participation can help individuals adapt to old age, adapt to changes in social roles, establish and develop new social interactions and social networks, and thus enhance subjective wellbeing. It has been found that people with significant hearing problems are three times more likely to drop out of leisure activities than those with good hearing (Mikkola et al., 2016), suggesting that older people’s hearing condition affects their participation in social activities. It has also been shown that all types of community activities can significantly reduce the risk of depression in older people (Li et al., 2020). Therefore, the impact of hearing status on depression may be acting through social engagement. Active social participation has a protective effect on aging in older adults. For example, resocialization theory suggests that active participation in various social activities can come to maintain and enhance social integration, reduce social isolation, and promote the development and improvement of the self. The findings of this study revealed a positive association between social participation and both functional ability and depressive symptoms among older adults. Specifically, greater engagement in social activities was associated with better daily living performance and lower levels of depression. These results highlight the salience of social participation for the mental health and wellbeing of older adults, with evidence suggesting that certain types of social participation may confer greater benefits than others (Zhang and Zhang, 2016). Older adults with hearing loss often opt to withdraw from social situations and activities that pose communication challenges, resulting in reduced social participation. Drawing on stress and coping theory, hearing impairment is regarded as an enduring stressor in the elderly, given the age-related decline in physiological functioning that underlies the condition. For older adults, it is more strenuous and difficult to choose problem-centered strategies that rely on exercise to restore their hearing abilities (Holmes and Rahe, 1967). The choice of avoidance strategies is more common in life by reducing one’s social interactions with others and thus reducing the inconvenience of communication. Studies have shown that the use of avoidance strategies usually leads to the deterioration of the individual’s physical and mental condition (Connor-Smith and Flachsbart, 2007). Social-emotional selectivity theory states that reduced social participation can also cause negative emotions such as anxiety, loneliness, and depression in older adults, and that reduced social participation may be an antecedent variable leading to depression. However, previous studies have shown that elevated negative affect such as depression and anxiety also cause decreased social participation, that depressive symptoms may be an antecedent variable of decreased social engagement, and that the correlation between decreased social engagement and depression is unclear (Croezen et al., 2015). Based on socioemotional selectivity theory, the present study tends to suggest that impaired hearing decreases social engagement in older adults and causes their depressed mood, that social engagement is an antecedent variable of depressive symptoms, and that social engagement plays an important mediating role in the interaction between hearing impairment and depressive symptoms.

In summary, with the aging of society becoming an increasingly pressing issue, there is growing concern regarding the physical and mental health of older adults. There exists a robust relationship between hearing impairment, depressive symptoms, and social participation among older adults. However, most of the previous studies were correlational studies, which did not clarify the degree of interaction between variables and the pathway of hearing impairment on depressive symptoms. On the other hand, most of the previous studies used cross-sectional studies, but it may be more meaningful to study the developmental trends and the inter-temporal effects of variables in aging older adults. Therefore, in this study, based on the combination of previous studies, we analyzed the longitudinal associations and directions of effects of hearing impairment, social participation, and depressive symptoms in older adults using cross-lagged models through a longitudinal study design, and developed structural equation models to explore the relationships between hearing impairment and depressive symptoms in older adults and their mechanisms of effects, and propose the hypotheses: (1) depressive symptoms and hearing impairment are co-morbid and they influence each other; (2) social participation is the antecedent of depressive symptoms; (3) hearing impairment in the elderly has an impact on depressive symptoms through the mediating role of social participation. Our study reveals mechanisms linking hearing impairment and depressive symptoms and provides theoretical support for the development of interventions to improve depressive symptoms in older people.

## 2. Materials and methods

### 2.1. Source of data

The China Health and Retirement Longitudinal Study (CHARLS) is a nationwide longitudinal survey administered by the National Development Institute of Peking University, aimed at gathering high-quality microdata from individuals aged 45 and above, as well as households, that are representative of the entire population in China (Zhao et al., 2014). CHARLS aims to study the aging process of China’s population. The baseline survey was conducted in 2011 and covered about 150 county-level units, 450 village-level units, and 17,000 individuals from 10,000 households, with biennial follow-up surveys conducted subsequently. The survey boasts a high response rate, with over 80% of participants agreeing to take part in the interviews, and the data obtained is of exceptional quality.

### 2.2. Participants

Two waves of data from CHARLS 2013 (T1) and 2018 (T2) were mainly used in this study. In the survey data, samples with more missing data, regular responses, logical errors and age below 60 years were excluded, and the final sample of 3,980 was screened. Using 2013 as the baseline data, the basic profiles of the subjects were: 2,121 (53.30%) males and 1,859 (46.70%) females. The subjects’ ages ranged from 60 to 93, with an average age of 66.16 ± 5.19. The subjects’ education averaged elementary school education, including 1,038 (26.1%) with less than elementary school, 881 (22.1%) with elementary school, 1,727 (43.3%) with secondary school education, and 338 (8.5%) with college education and above.

### 2.3. Research tools

#### 2.3.1. Measurement of hearing impairment

The DA039 question in the CHARLS questionnaire was used as a measure of hearing impairment, “How is your hearing?” (If you wear hearing aids regularly, how do you hear when you wear them? If you do not wear hearing aids regularly, how do you hear when you do not wear hearing aids?). A total of 1–5 scale, the greater the score, the more pronounced the hearing impairment and the poorer the hearing ability.

#### 2.3.2. Measurement of social participation

The DA056 question “Did you do the following social activities in the past month (multiple choice)” and the DA057 question “How often did you participate in social activities” were used. The higher the score, the higher the frequency of social participation of the elderly.

#### 2.3.3. Measurement of depressive symptoms

The CHARLS survey embedded the CES-D10 short form into the questionnaire (DC009-018) and used 10 questions to measure the depressive status of the respondents, namely: “I am bothered by some small things,” “I have difficulty concentrating when I have a hard time concentrating when doing things” and so on. In this study, the severity of depressive symptoms was assessed using a scale consisting of items scored on a 4-point scale. Reverse coding was applied to positive questions to ensure consistency. The sum of the item scores was used to calculate the total score of the scale, where higher scores indicated more severe symptoms. The reliability of the scale was evaluated using the Cronbach’s alpha coefficient, which was found to be 0.71 for the 2013 data and 0.78 for the 2018 data, indicating relatively stable reliability across time points.

### 2.4. Data processing

Descriptive statistics and partial correlation analysis were performed using SPSS 23.0, cross-lagged analysis and modeling of latent factor structural equations were performed using Mplus 7.4, and tests for mediating effects and estimation of confidence intervals were performed using the Bootstrap method with 5,000 replicate samples.

## 3. Results

### 3.1. Common method bias

To examine the potential presence of common method bias in this study, a statistical procedure known as Harman’s one-way test was conducted, as recommended by Zhou and Long (2004) for identifying the existence of a single dominant factor that can explain most of the variance in the data. The results of the analysis revealed that six factors had eigenvalues greater than 1, indicating that multiple underlying factors are responsible for the variation in the data. Additionally, the first factor extracted from the analysis accounted for only 23.85% of the total variance, which is below the widely accepted threshold of 40%, thereby indicating that there is no significant common method bias in this study.

### 3.2. Descriptive statistics and correlation analysis

Controlling for demographic variables, the results of the partial correlation analysis indicated significant associations between hearing impairment, social participation, and depressive symptoms. The findings indicated that hearing impairment was negatively correlated with social participation and positively correlated with depressive symptoms at each time point. Moreover, social participation was negatively correlated with depressive symptoms at each time point.

Between the two measures, T1 and T2 counterparts were positively correlated, T1 hearing impairment was negatively correlated with T2 social participation (*r* = −0.08, *p* < 0.001) and T2 depressive symptoms (*r* = 0.14, *p* < 0.001), and T1 social participation and T2 depressive symptoms were negatively correlated (*r* = −0.05, *p* < 0.001). Paired *t*-tests on data from both waves showed that for the 2013 baseline, at 5 years, older adults had significantly higher hearing impairment (*D* = −0.14, *t* = −8.62, *p* < 0.001), depressive symptoms (*D* = 0.34, *t* = 8.42, *p* < 0.001), and significantly lower social participation (*D* = −1.25, *t* = −12.33, *p* < 0.001), and see Table 1 for a summary of the results.

**TABLE 1:** Descriptive statistics and correlations for each dimension.

Variable dimension	*M* (*SD*)	*D*	*t*	1	2	3	4	5	6
1. T1 hearing impairment	3.61 (0.96)	−0.14	−8.62***	–	−0.07***	0.19***	0.43***	−0.08***	0.14***
2. T1 social participation	1.97 (2.34)	0.34	8.42***		–	−0.09***	−0.06***	0.41***	−0.05***
3. T1 depressive symptoms	17.50 (5.34)	−1.25	−12.33***			–	0.17***	−0.05***	0.43***
4. T2 hearing impairment	3.75 (0.91)						–	−0.07***	0.18***
5. T2 social participation	1.63 (2.21)							–	−0.07***
6. T2 depressive symptoms	18.75 (6.27)								–

*p < 0.05, **p < 0.01, ***p < 0.001.

*D* is the mean of the difference between T1 and T2.

Overall, these results suggest that hearing impairment may be associated with decreased social participation and increased depressive symptoms in older adults, and that these issues may worsen over time. Interventions aimed at improving hearing and increasing social participation may be beneficial in promoting mental health and wellbeing in older adults.

### 3.3. Cross-lagged analysis

The results showed that the model was a supersaturated model, with a chi-square statistic of 0 and 0 degrees of freedom, indicating that the model was overidentified. The fit indices, including the comparative fit index (CFI = 1.00), root mean square error of approximation (RMSEA = 0.00), and standardized root mean square residual (SRMR = 0.00), indicated that the model fit the data well.

**FIGURE 1:**
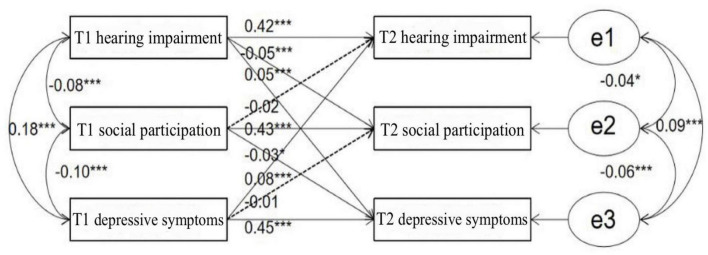
Cross-lagged analysis. *p < 0.05, **p < 0.01, ***p < 0.001.

The path coefficients showed that all correlations between T1 and T2 variables were significant, providing support for the autoregressive cross-lagged design hypothesis. T1 hearing impairment significantly positively predicted T2 hearing impairment (β = 0.42, *p* < 0.001) and T2 depressive symptoms (β = 0.05, *p* < 0.001) and significantly negatively predicted T2 social participation (β = −0.05, *p* < 0.001). T1 social participation significantly positively predicted T2 social participation (β = 0.43, *p* < 0.001) and negatively predicted T2 depressive symptoms (β = −0.03, *p* = 0.05). T1 depressive symptoms significantly positively predicted T2 hearing impairment (β = 0.05, *p* < 0.001) and T2 depressive symptoms (β = 0.45, *p* < 0.001). However, T1 social participation did not significantly predict T2 hearing impairment (β = −0.02, *p* = 0.27), and T1 depressive symptoms did not significantly predict T2 social participation (β = −0.01, *p* = 0.63) and the results are shown in Figure 1.

Based on the results of the above data, we can see that there is a cumulative effect of risk for hearing impairment, depressive symptoms, and social participation in older adults, which become progressively more severe with age. Hearing impairment and depressive symptoms are mutually predictive and can be considered co-morbid, with hearing impairment leading to increased depressive symptoms, and depressive symptoms leading to hearing loss. Hearing impairment is an antecedent variable of social participation, which in turn is an antecedent variable of depressive symptoms. Hence, the impact of hearing impairment on depressive symptoms may involve both direct and indirect mechanisms, and social participation could potentially serve as a mediator in the association between depressive symptoms and hearing impairment.

### 3.4. Mediational analysis

**FIGURE 2:**
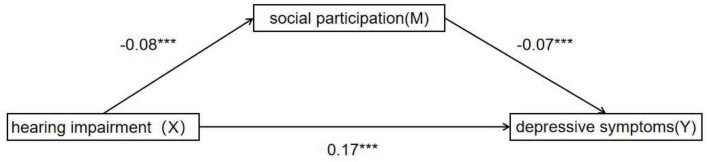
Mediation analysis. *p < 0.05, **p < 0.01, ***p < 0.001.

We utilized structural equation modeling on the 2018 CHARLS data to examine the mediating role of social participation in the relationship between hearing impairment and depressive symptoms in older adults. The results revealed that hearing impairment in older adults was significantly and positively associated with depressive symptoms (β = 0.17, *p* < 0.001). The 95% confidence interval (CI) for the path coefficients of each variable did not contain 0, indicating a significant relationship between hearing impairment and depressive symptoms (see Figure 2 and Table 2). Moreover, social participation partially mediated the relationship between hearing impairment and depressive symptoms, accounting for 5.56% of the total effect size. The indirect effect value was 0.01, 95% CI [0, 0.01], which was statistically significant.

**TABLE 2:** Mediational analysis.

Variable	β	Effect size	Proportions of effect	95% CI
Hearing impairment(X)-depressive symptoms(Y)	0.17			[0.14, 0.20]
Hearing impairment(X)-social participation(M)	−0.08			[−0.11, −0.04]
Social participation(M)-depressive symptoms(Y)	−0.07			[−0.11, −0.05]
Direct effect		0.17	94.44%	[0.14, 0.20]
Indirect effect		0.01	5.56%	[0, 0.01]
Total effect		0.18		[0.14, 0.20]

Further analysis using the Bootstrap method with 5,000 replicate samples showed that the total effect value of communication disorders in older adults on depressive symptoms was 0.18, 95% CI [0.14, 0.20]. The direct effect value was 0.17, 95% CI [0.14, 0.20], accounting for 94.44% of the total effect size. These results suggest that the direct impact of hearing impairment on depressive symptoms is substantial, while the mediating role of social participation is relatively small.

Two distinct pathways, direct and indirect, were found to be involved in the relationship between hearing impairment and depressive symptoms in older adults, according to the results. Furthermore, the indirect pathway is mediated by social participation, meaning that social participation serves as a mediating variable between depressive symptoms and hearing impairment.

## 4. Discussion

In the present study, utilizing data from the 2013 and 2018 waves of the CHARLS database, a cross-lagged analysis was conducted to examine the associations among hearing impairment, depressive symptoms, and social participation in older adults. Results indicated significant correlations between these variables across the two time points, with notable declines observed in hearing impairment, depressive symptoms, and social participation in 2018 relative to 2013. Notably, hearing impairment was found to precede reductions in social engagement, which in turn predicted increases in depressive symptoms. Moreover, hearing impairment and depressive symptoms were identified as interacting as causal factors. The utilization of structural equation modeling allowed for further exploration of these relationships, with analysis conducted on 2018 data indicating that social participation served as a mediating factor in the connection between depressive symptoms and hearing impairment among the elderly. The results of this study offer novel perspectives on the connections among hearing impairment, depressive symptoms, and social participation in older adults. Our findings highlight the importance of prioritizing hearing impairment in older populations and implementing interventions to enhance mental health and social participation as a means of reducing the likelihood of depression in older adults. Theoretical and practical implications for future research are discussed.

The present study found that hearing impairment in older adults positively predicted depressive symptoms, which is in line with stress and coping theory, then hearing impairment is perceived by older adults as a potential stressor that continues to undermine their mental health. This is also consistent with the findings of Deary et al. (2003) and Siupsinskiene et al. (2011) that the severity of hearing problems can lead to increased anxiety, depression, and other negative emotions. On the other hand, depressive symptoms in the elderly can also lead to hearing loss. Depressive symptoms, as a serious psychological problem, can lead to reduced psychological flexibility and affect the management of somatic diseases in individuals, mainly in terms of poor compliance with medication, untimely visits to the doctor, and reduced willingness to seek treatment. Depressive symptoms and hearing impairment in the elderly are causal, occurring simultaneously and interacting with each other. As we age, the function of the auditory system deteriorates and hearing loss often occurs in conjunction with other diseases and is often a concomitant symptom of multiple diseases. Depression can be triggered or exacerbated by the reduced function and lifestyle changes caused by somatic diseases, while the psychological and systemic dysregulation caused by depression can accelerate the deterioration of somatic diseases, patients with geriatric depression often have significant cognitive and emotional impairments, such as memory loss, inattention, and emotional instability (Wang, 2020), forming a vicious circle that exacerbates physical and mental impairment.

Hearing impairment in older adults negatively predicts social engagement and social engagement negatively predicts depressive symptoms, and this predictive relationship is unidirectional, with hearing impairment as an antecedent variable of social engagement and social engagement as an antecedent variable of depressive symptoms. Older adults develop hearing impairment due to physical decline or disease, which affects various activities in daily life (Farley et al., 2006). Then, in order to avoid this social inconvenience, older adults, according to stress and coping theory, are forced to adopt passive avoidance strategies due to the degeneration of somatic functions and actively reduce social participation thus reducing the communication inconvenience caused by hearing loss, and avoidance strategies often lead to the deterioration of the individual’s physical and mental condition (Connor-Smith and Flachsbart, 2007). According to self-efficacy theory, due to the decline of physical functions, older adults find themselves gradually losing control of their physical functions, such as language skills, which leads to a sense of powerlessness and loss of interest in life, and gradually decreases their self-efficacy for social participation and social communication, this sense of powerlessness can affect older adults’ feelings of loneliness and depression, which seriously affects their quality of life. In contrast, depressive symptoms do not lead to reduced social engagement, as well as reduced social engagement does not affect hearing levels in older adults (Li et al., 2020). Social participation is often used as an intervention for depressive symptoms, and individuals may choose to alleviate depression by increasing communication with others and increasing social participation when they become depressed. Therefore, although depressive symptoms affect an individual’s psychological flexibility, in order to alleviate this loneliness and depression, individuals will instead maintain or even increase their social involvement, but which coping style an individual will exhibit will vary depending on social, environmental, and individual reasons. So social engagement is an antecedent variable of depressive symptoms, but depressive symptoms are not an antecedent variable of social engagement. Hearing impairment in the elderly is a decline in auditory function due to aging and degenerative lesions of the auditory organs, and this change is irreversible and at most slows down its decline, and is more affected by the individual’s somatic and psychological status and less by social interactions. For elderly people with hearing loss, their social participation is bound to decline. According to communication adaptation theory, the communication objectives of the hearing impaired may take methods to reduce the inconvenience of communication such as raising the voice, reducing the speed of speech and simplifying the content, but all these methods will increase the psychological cost of the communication object, and this increase in psychological cost will make the communication object reduce the frequency and time of communication with the communication impaired elderly, and reduce the frequency of social participation of the elderly (Baxter et al., 2002; Bute et al., 2007). Therefore, hearing impairment is an antecedent variable of social participation, but depressive symptoms are not an antecedent variable of social participation.

Utilizing path analysis and a mediated effects test with the 2018 data, our study found evidence for partial mediation between hearing impairment, social engagement, and depressive symptoms among older adults. This supports previous research that has demonstrated a strong association between these variables (Parr, 2007; Davidson et al., 2008). Drawing on stress and coping theory, our findings suggest that hearing impairment serves as a psychological stressor for older adults, directly impacting their physical and mental health. As a result of physical limitations, older adults may adopt passive avoidance coping strategies, reducing social participation and mitigating the inconvenience associated with hearing loss (Holmes and Rahe, 1967). However, social participation is a crucial factor in the development of depressive symptoms, and declining social engagement may exacerbate depressive symptoms among older adults. Our cross-lagged analysis revealed bidirectional associations between hearing impairment and depressive symptoms, forming a vicious cycle in which social participation mediates the relationship between the two and exacerbates functional impairment. In line with these findings, our study validated a partial mediation model with social participation as a mediating variable, wherein the severity of hearing impairment in older adults influences depressive symptoms by reducing social participation. These results have important implications for the development of interventions aimed at reducing depressive symptoms among older adults through the promotion of social engagement and the mitigation of hearing impairment.

This study used a combination of a longitudinal study of database data and structural equation modeling to comprehensively and thoroughly examine the relationships among variables such as communication disorders, social participation, and depressive symptoms in older adults. The study was based on a large amount of data, used a longitudinal study, analyzed using cross-lagged models, and drew statistically significant conclusions and explained the findings using stress coping theory, self-efficacy theory, communication adaptation theory and environmental adaptation theory. However, three shortcomings remain in this study. Firstly, the study data was small and separated by a long period of time, which did not allow for a detailed analysis of the dynamics of each variable. Secondly, although the study identified the effects of communication barriers, social participation and loneliness on depressive symptoms in older adults, no further intervention experiments were conducted to prove the findings and suggest practical interventions. Furthermore, variables such as communication impairment and social engagement are usually uncontrollable and irreversible, which are inevitable results of aging in the elderly, so the relevance of this study is weakened.

The results of this paper reveal how hearing status affects depression levels in older people and have implications for the prevention or reduction of depressive symptoms in older people with hearing impairment. Firstly, older people should have their hearing checked regularly to enable early detection, intervention and treatment. When hearing impairment is detected, timely intervention, such as the use of hearing aids, should be carried out to make the most of the residual hearing of older people and improve their standard of living. Secondly, engage in appropriate social participation. Social participation can provide opportunities for older people to try out different roles and help establish new social roles and social interactions, thereby alleviating mental health problems (Mikkola et al., 2016). In addition, children should be aware of the hearing status of older people when communicating with them. When communicating with older people with hearing impairment, children should be careful to slow down their speech, be patient and reassuring, give positive psychological support and encourage older people to get out of the house and participate in more social activities. Finally, provide policy support for the hearing health and mental health of the elderly. For example, setting strict maximum limits for noise in the community, reducing the decibels of noise in the community and creating a good low-noise environment; optimizing medical services so that older people can apply for hearing aids with relative ease and opening relevant mental health consultation rooms; and organizing various community group activities every month in the community and encouraging older people to participate actively in them.

Future research could explore the language comprehension aspect of communication disorders, distinguish somatic aging from cognitive aging, and explore whether the decline in language comprehension due to cognitive aging also affects social interactions and depressive symptoms in older adults. Secondly, to examine whether the type and quality of social participation also change with age, and thus whether they also affect depressive symptoms in older adults. Finally, researchers can combine other research methods in psychology, such as experimental methods and interviews, to obtain richer data and to conduct intervention experiments that can practically reduce depressive symptoms in older adults.

## 5. Conclusion

Hearing impairment and depressive symptoms in older adults are mutually causal and co-morbid, with hearing impairment being an antecedent variable of social participation and social participation being an antecedent variable of depressive symptoms. Hearing impairment in older adults not only directly affects their depressive symptoms, but also indirectly affects their depressive symptoms through the mediating role of social participation.

## Data availability statement

The original contributions presented in this study are included in the article/supplementary material, further inquiries can be directed to the corresponding author.

## Author contributions

HZ and XW collected the data and wrote the manuscript. YS wrote the manuscript and revised the manuscript. All authors contributed to the article and approved the submitted version.
